# Physically Active Lifestyle Is Associated With Attenuation of Hippocampal Dysfunction in Cognitively Intact Older Adults

**DOI:** 10.3389/fnagi.2021.720990

**Published:** 2021-10-06

**Authors:** Tamir Eisenstein, Nir Giladi, Talma Hendler, Ofer Havakuk, Yulia Lerner

**Affiliations:** ^1^Sackler Faculty of Medicine, Tel Aviv University, Tel Aviv, Israel; ^2^Sagol Brain Institute, Tel Aviv Sourasky Medical Center, Tel Aviv, Israel; ^3^Department of Neurology, Tel Aviv Sourasky Medical Center, Tel Aviv, Israel; ^4^Sagol School of Neuroscience, Tel Aviv University, Tel Aviv, Israel; ^5^School of Psychological Sciences, Tel Aviv University, Tel Aviv, Israel; ^6^Department of Cardiology, Tel Aviv Sourasky Medical Center, Tel Aviv, Israel

**Keywords:** hippocampus, aerobic exercise, neuroimaging, memory, functional connectivity

## Abstract

Alterations in hippocampal function have been shown in older adults, which are expressed as changes in hippocampal activity and connectivity. While hippocampal activation during memory demands has been demonstrated to decrease with age, some older individuals present increased activity, or hyperactivity, of the hippocampus which is associated with increased neuropathology and poor memory function. In addition, lower functional coherence between the hippocampus and core hubs of the default mode network (DMN), namely, the posteromedial and medial prefrontal cortices, as well as increased local intrahippocampal connectivity, were also demonstrated in cognitively intact older adults. Aerobic exercise has been shown to elicit neuroprotective effects on hippocampal structure and vasculature in aging, and improvements in cardiorespiratory fitness have been suggested to mediate these exercise-related effects. However, how these lifestyle factors relate to hippocampal function is not clear. Fifty-two cognitively intact older adults (aged 65–80 years) have been recruited and divided into physically active (*n* = 29) or non-active (*n* = 23) groups based on their aerobic activity lifestyle habits. Participants underwent resting-state and task-based fMRI experiments which included an associative memory encoding paradigm followed by a post-scan memory recognition test. In addition, 44 participants also performed cardiopulmonary exercise tests to evaluate cardiorespiratory fitness by measuring peak oxygen consumption (Vo_2_peak). While both groups demonstrated increased anterior hippocampal activation during memory encoding, a physically active lifestyle was associated with significantly lower activity level and higher memory performance in the recognition task. In addition, the physically active group also demonstrated higher functional connectivity of the anterior and posterior hippocampi with the core hubs of the DMN and lower local intra-hippocampal connectivity within and between hemispheres. Vo_2_peak was negatively associated with the hippocampal activation level and demonstrated a positive correlation with hippocampal-DMN connectivity. According to these findings, an aerobically active lifestyle may be associated with attenuation of hippocampal dysfunction in cognitively intact older adults.

## Introduction

Episodic memory, the ability to encode, consolidate, and retrieve past experiences and events, is one of the most affected cognitive abilities during aging (Nyberg et al., 2012). The hippocampus is a brain region that plays a key role in episodic memory processes (Squire and Zola-Morgan, 1991; Squire and Wixted, 2011; Insausti et al., 2013), through anatomical and functional interactions with cortical and subcortical regions across the brain (Ranganath and Ritchey, 2012; Cooper and Ritchey, 2019). The hippocampus presents distinct structural and functional connections along its longitudinal axis with its anterior part being more connected to anterior temporal and ventromedial prefrontal regions and its posterior part more linked to posterior-medial cortices and hubs of the default mode network (DMN) such as the posterior cingulate cortex (Ranganath and Ritchey, 2012). Moreover, from a cognitive perspective, the anterior hippocampus has been shown to be more associated with memory encoding while its posterior part has been more related to retrieval (Grady, 2020).

Older adults have been demonstrated to exhibit age-related alterations in hippocampal function. Hippocampal activity patterns during memory demands have been shown to generally decrease with age (Mormino et al., 2012; Ta et al., 2012; Nyberg et al., 2019). Furthermore, this age-related hypoactivity pattern was suggested to be region and process selective, affecting more pronouncedly the anterior part of the hippocampus during encoding (Ta et al., 2012; Nyberg et al., 2019). However, some older individuals demonstrate abnormally increased hippocampal activation during memory encoding and retrieval compared with both old and younger counterparts, also referred to as hippocampal hyperactivity. In addition, although this phenomenon has been observed in both healthy aging and prodromal stages of Alzheimer's disease (AD), such as mild cognitive impairment (MCI), it is not clear whether this activity pattern represents a compensatory effect or a dysfunctional pathological consequence.

While increased hippocampal activity or hyperactivity has been generally demonstrated to be associated with poorer neurobiological and cognitive outcomes in cognitively healthy older adults, it has been both negatively and positively linked with cognitive and clinical outcomes in patients with MCI (Kircher et al., 2007; Clement and Belleville, 2010; Bakker et al., 2012; Huijbers et al., 2015; Eisenstein et al., 2020). For example, increased left hippocampal activation during memory encoding in MCI was correlated with better memory performance and cognitive-clinical status in some studies (Kircher et al., 2007; Clement and Belleville, 2010), while others found it to be associated with poorer memory performance and increased Aβ burden (Bakker et al., 2012; Huijbers et al., 2015).

In addition to altered activation levels, changes in the resting-state functional connectivity of the hippocampus have also been documented to take place during aging. The hippocampus constitutes a part of the medial temporal subsystem of the DMN and is functionally and anatomically connected with regions comprising this network (Andrews-Hanna et al., 2010). However, the study suggests that with increasing age, the hippocampal activity becomes less coherent with the activity of distant brain regions such as major hubs of the DMN including the posteromedial and medial prefrontal cortices (or decreased hippocampal-DMN resting-state functional connectivity) and more locally connected within itself (or increased intra-hippocampal resting-state functional connectivity) (Salami et al., 2014; Damoiseaux et al., 2016; Harrison et al., 2019). Furthermore, this increased local connectivity was found to be associated with increased AD pathology burden in distinct memory networks and poorer episodic memory performance.

While neurobiological alterations have been well documented in the hippocampus during aging, several lifestyle factors have been proposed to attenuate this age-related deterioration, one of which is physical activity. The physically active lifestyle has been associated with the prevention of cognitive decline and the risk of AD and dementia (Rovio et al., 2005; Larson et al., 2006; Andel et al., 2008). Aerobic exercise has been associated with hippocampal neuroprotective effects in both animal models (van Praag et al., 1999, 2005; Vaynman et al., 2004; Bednarczyk et al., 2009; Van der Borght et al., 2009) and healthy human subjects (Erickson et al., 2011; Maass et al., 2015; Kleemeyer et al., 2016). However, studies examining the effect of physical exercise on the hippocampus in older adults had largely focused on structural characteristics, and to our knowledge, no previous work had focused specifically on the relationship between the aerobically active lifestyle and hippocampal function in human aging.

Several factors have been suggested to potentially mediate the effects of aerobic exercise on the brain. Peak oxygen consumption (Vo_2_peak) is perhaps the most studied physiological correlate of aerobic/cardiorespiratory fitness in this context, and it reflects the peak metabolic rate of the body in generating adenosine triphosphate molecules through aerobic metabolism (Wilmore et al., 2008). Previous studies that demonstrated neuroprotective effects of aerobic exercise intervention in older adults also found favorable structural and cerebrovascular hippocampal changes to correlate with improved Vo_2_peak (Erickson et al., 2011; Maass et al., 2015; Kleemeyer et al., 2016). However, to our knowledge, no study to date specifically examined the relationship between Vo_2_peak and age-related hippocampal dysfunction patterns.

This study aimed to address this gap by investigating the relationship between aerobic exercise and hippocampal function of lifestyle during both resting-state and active memory demands in cognitively intact older adults. In addition, we aimed to examine whether the extent of these trends may be associated with the level of Vo_2_peak. We first aimed to examine the activity levels of the anterior hippocampus during the associative memory encoding task, since this hippocampal subpart has been shown to be more involved in memory encoding processing (Grady, 2020). The associative memory paradigm was chosen as this form of episodic memory has been shown to be highly sensitive to aging compared with item memory and relies greatly on hippocampal function (Old and Naveh-Benjamin, 2008). Then, we aimed to investigate both remote and local resting-state functional connectivity patterns of both anterior and posterior hippocampal subparts. Distant hippocampal resting-state functional connectivity was examined with the two core hubs of the DMN, i.e., the posteromedial and medial prefrontal cortices (hippocampal-DMN), and intra-hippocampal resting-state functional connectivity patterns were examined between bilateral anterior and posterior hippocampi. We then examined whether physically active individuals may demonstrate distinct patterns of hippocampal activity and functional connectivity compared with sedentary individuals, and whether it may explain differences in memory performance between the two groups. Finally, we investigated the correlations between Vo_2_peak and all hippocampal and memory measures. According to the previous findings in the literature, we hypothesized that physically active older adults will demonstrate lower anterior hippocampal activity during memory encoding, higher memory performance, higher distant functional connectivity with the cortical DMN hubs, and lower within hippocampal functional correlation. In accordance, we hypothesized that Vo_2_peak, as a potential mediator of aerobic exercise, will demonstrate dose-response correlations with hippocampal function and memory measures in the same directions as physically active lifestyle.

## Materials and Methods

### Participants

Fifty-two older adults aged 65–80 years were recruited for this study (22 women/30 men). All participants were recruited from the community via an online advert on the Israeli Ministry of Health research website, social media, and by “word-of-mouth.” Participants were fluent Hebrew speakers and reported no current or previous neuropsychiatric disorders (e.g., Parkinson's disease, brain tumor, head injury, transient memory loss, stroke, subjective cognitive/memory decline, psychosis, bipolar disorder, and depressive symptoms) or any other current significant uncontrolled and unbalanced medical illness (e.g., cardiac or vascular disease, hypertension, type II diabetes, cancer, and autoimmune syndromes). Nine participants reported being diagnosed with hypertension; however, they were included in this study since their blood pressure was controlled and balanced. This study was approved by the Human Studies Committee of Tel Aviv Sourasky Medical Center (TASMC), and all participants provided written informed consent to participate in this study.

### Assessment of Aerobic Activity Lifestyle Habits

The current aerobically active lifestyle of participants was assessed using a background interview that examined background characteristics and the physical exercise habits of the participants during the last year. The interview was administered face-to-face on the first assessment day. Specifically, participants were asked regarding the amount of weekly exercise-oriented activity sessions of some types of aerobic exercises (i.e., walking, running, cycling, swimming, elliptical/cross-training gym machines, etc.) or “*How often during the week did you participate in leisure time aerobic activity for the purpose of sporting or exercise that lasts at least 20 min in the passing year?”* Then, based on their reported activity patterns, the participants were divided into two groups, namely, active (*n* = 29) and non-active (*n* = 23). Frequency of twice-a-week or higher was set as the cutoff for active lifestyle based on previous methodologies (Rovio et al., 2005, 2010), findings (Liu-Ambrose et al., 2012), and recommendations (Voelcker-Rehage et al., 2010; Petersen et al., 2018). In addition, since we used a subjective measure of physical activity, using a cutoff of several times per week may help to decrease potential information bias from the reports of participants on their true activity levels. Also, regarding the potential of self-selection bias of volunteering in this study, it is important to note that the fraction of the active group out of the whole sample (56%) was similar to a recent report on the aerobically active lifestyle habits among older adults in the areal county from which participants were recruited (53.5% who reported being active) (Central Bureau of Statistics of Israel, 2021).

### Vo_2_Peak Measurement

Out of 52 participants, 44 participants (16 women/28 men) also underwent a graded maximal cardiopulmonary exercise test performed on a cycle ergometer (Ergoselect 100, Ergoline, GmBH, Germany) to evaluate Vo_2_peak. Assessments were conducted at the Non-Invasive Cardiology Outpatient Clinic at TASMC. Tests were performed using a metabolic cart (ZAN, nSpire Health Inc., Longmont, Colorado) while continuously measuring breath-by-breath minute ventilation, carbon dioxide production (Vco_2_), oxygen consumption (Vo_2_), and respiratory exchange ratio (RER). In addition, a 12-lead electrocardiograph, non-invasive arterial saturation, heart rate, and blood pressure were monitored continuously. All tests were supervised by a cardiologist and an exercise physiologist. An automated computerized ramp protocol was used to increase the exercise intensity by 10 W/min for women and 15 W/min for men, while participants were asked to maintain a constant velocity of 60 revolutions per minute. All tests were performed until volitional exhaustion, and no adverse events or medical symptoms were reported. An RER value of ≥1.1 was used as the indication for a satisfactory effort level during test (Balady et al., 2010) and was demonstrated in all examinations. Plateau in Vo_2_ was not evident in any participant; therefore, the highest Vo_2_ demonstrated in each test was considered as a Vo_2_peak. The highest average Vo_2_ value recorded during an intensity interval (2/2.5 W increment for women and men, respectively) was considered as the Vo_2_peak value obtained from the procedure and that was used in further analyses.

### Experimental Procedure and Stimuli

#### Inside the MRI Scanner

**Figure 1:**
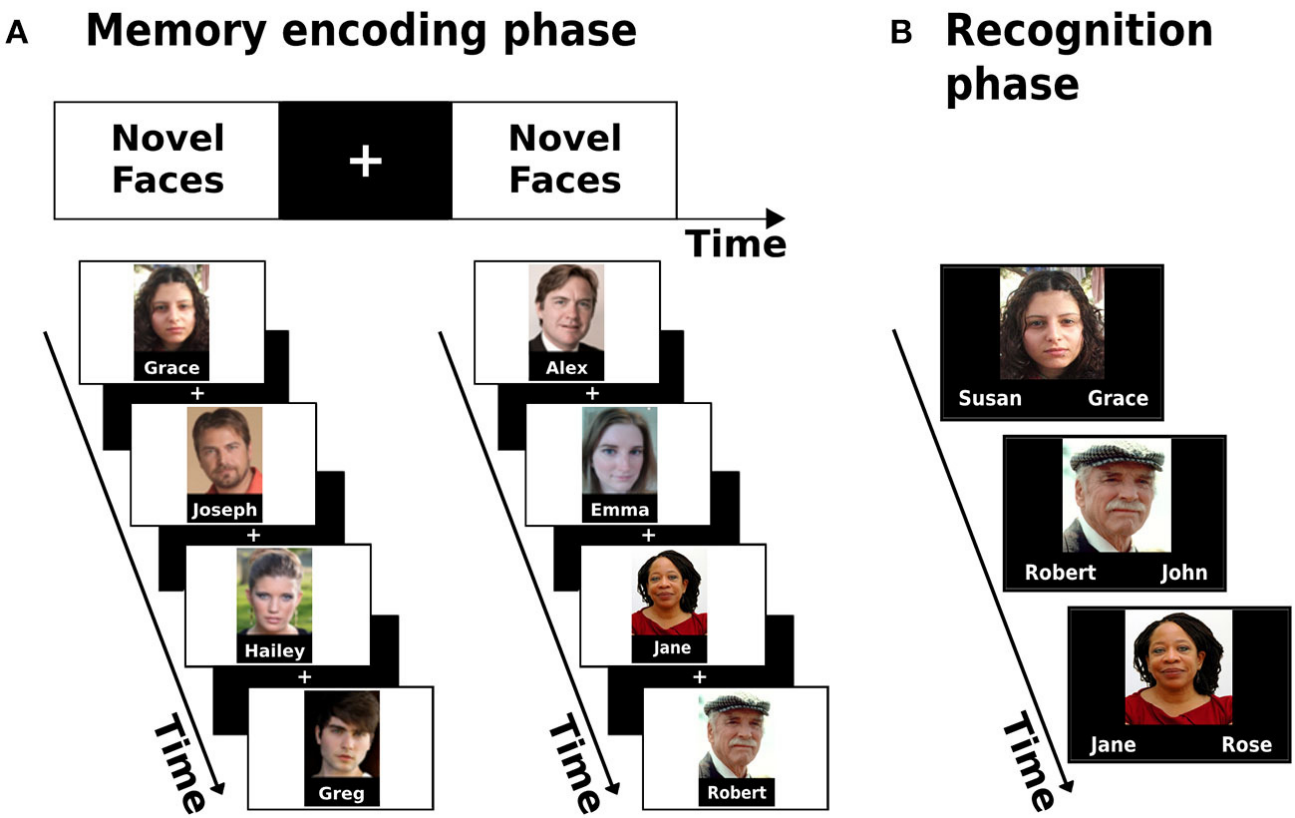
Associative memory encoding paradigm conducted in the scanner **(A)** and the post-scan memory recognition task **(B)**.

A face-name associative memory encoding task based on the classic paradigm by Sperling et al. (2001, 2003) was used to evaluate the hippocampal function. During scanning, participants were shown novel images of non-famous faces, each face paired with a fictional name (Figure 1A). Participants were asked to memorize which name was coupled with each face and to subjectively decide (by pressing a button) whether the name “fits” or not to the face. This subjective decision has been shown to enhance associative encoding (Sperling et al., 2003). Participants performed one run of a block-design picture-viewing paradigm consisted of 8 blocks with 4 faces presented in each block (overall 32 faces). Between blocks and consecutive in-block images, participants were shown “resting” fixation blocks of a white cross in the middle of a black background. Before the scanning session, participants underwent a familiarization practice with the task to minimize novel task learning effects which could bias the neurocognitive outcome. Each stimuli block lasted for 21 s, while the overall run lasted 5:06 min.

#### Outside the Scanner–Memory Performance Evaluation

Following the acquisition phase in the scanner, participants performed a two-alternative forced-choice recognition task. During the task, participants were shown images of all 32 faces presented during the encoding task and were asked to decide between two options which name was paired with each face (Figure 1B). The scores on the task were later used as the measure of memory performance.

### MRI Data Acquisition

MRI scanning was performed at TASMC on a 3 T Siemens system (MAGNETOM Prisma, Germany). High resolution, anatomical T1-weighted images (voxel size = 1 × 1 × 1 mm) were acquired with a magnetization prepared rapid acquisition gradient-echo protocol with 176 contiguous slices using the following parameters: field of view (FOV) = 256 mm; matrix size = 256 × 192; repetition time (TR) = 1,740 ms; echo time (TE) = 2.74 ms, inversion time (TI) = 976 ms, flip angle (FA) = 8°. These anatomical volumes were used for structural segmentation and co-registration with functional images. Blood oxygenation level-dependent functional MRI was acquired with T2*-weighted imaging. The memory encoding task was conducted using the following parameters: 102 TRs of 3,000 ms each; TE = 35 ms; FA = 90°; FOV = 220 mm; matrix size = 96 × 96; 44 slices, size = 2.3 × 2.3 × 3 mm, no gap (5:06 min run). The resting-state run was carried out with the same parameters with 120 TRs (6:00 min run).

To minimize head movements, the head of participants was stabilized with foam padding. MRI-compatible headphones (OPTOACTIVEtm) were used to considerably attenuate the scanner noise and communicate with the participants during the session. Designated software (Presentation^®^, Neurobehavioral Systems) was used for the presentation of visual stimuli.

### Functional MRI Analysis

#### Hippocampal Activation

**Figure 2:**
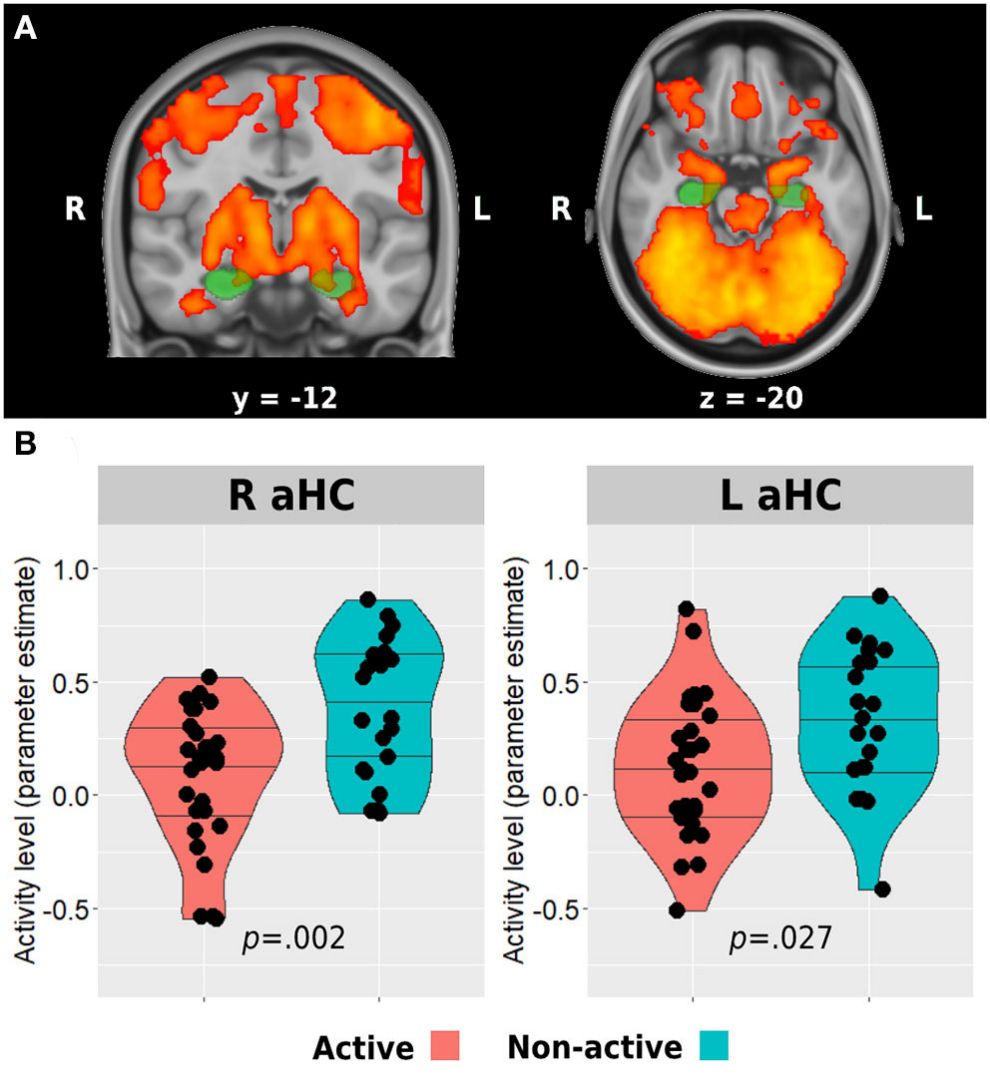
Anterior hippocampus activation during memory encoding. The whole sample mean activation during the task is presented in **(A)**. Green regions represent anatomical masks of anterior hippocampi, while activated voxels within these areas were used to extract the activity level for the between-group analysis presented in **(B)**. Horizontal lines in between-group plots represent 25, 50, and 75% quantiles. aHC, anterior hippocampus; L, left; R, right.

The fMRI analysis was carried out using the FEAT tool in FMRIB's Software Library 6.00 (FSL, www.fmrib.ox.ac.uk/fsl). The first five TRs of the functional data were discarded to allow steady-state magnetization. Registration of the functional data to the high-resolution structural images was carried out using boundary-based registration algorithm (Greve and Fischl, 2009). Registration of high-resolution structural to standard space (MNI152) was carried out using FLIRT (Jenkinson and Smith, 2001; Jenkinson et al., 2002) and then further refined using FNIRT non-linear registration. Motion correction of functional data was carried out using MCFLIRT (Jenkinson et al., 2002), brain removal using BET (Smith, 2002), spatial smoothing using a Gaussian kernel of 5-mm FWHM, grand-mean intensity normalization of the entire 4D dataset by using a single multiplicative factor, and high-pass temporal filtering was performed with a Gaussian-weighted least-squares straight-line fitting with a cutoff period of 100 s. Time-series statistical analysis (pre-whitening) was carried out using FILM with local autocorrelation correction (Woolrich et al., 2001). A first-level task regressor of interest was defined and convolved using the block onset times with a double-gamma hemodynamic response function and a temporal derivative regressor of the task timing. In addition, 24 nuisance motion regressors were added to each first-level model and included 6 standard motion parameters (i.e., 3 rotations and 3 translations), their temporal derivatives, and squares of all the above. Moreover, volumes with excessive head motion (predetermined as frame-wise-displacement value > 0.9 mm) were scrubbed by adding an additional regressor for each volume to be removed. The participants were removed and excluded from the group analysis if they had 30% or more of their volumes scrubbed out (one participant from the non-active group). *Z* statistic images were thresholded non-parametrically using clusters determined by *Z* > 3.1 and a corrected cluster significance threshold of *p* = 0.05. Group-level analysis was carried out using FLAME (FMRIB's Local Analysis of Mixed Effects) stage 1 (Beckmann et al., 2003; Woolrich et al., 2004; Woolrich, 2008). Group-level *Z* statistic images were thresholded non-parametrically using clusters determined by *Z* > 2.3 and a corrected cluster significance threshold of *p* = 0.05. Another participant from the non-active group did not go through the encoding run. The mean activation patterns observed during the encoding task of the entire sample were used to identify anterior hippocampal regions which demonstrated increased activity during the task. Then, a mask was created for these regions (for each hemisphere) by masking the activation map with anatomical anterior hippocampal masks from https://neurovault.org/collections/3731/ based on the study by Ritchey et al. (2015; Figure 2A). Then, we used the *Featquery* tool of FSL, which enables the interrogation of FEAT results within a specific mask, to extract the parameter estimates from the bilateral anterior hippocampal regions activated during the task. The parameter estimate values were then used to examine the differences in activation levels between the groups.

#### Hippocampal Resting-State Functional Connectivity

**Figure 3:**
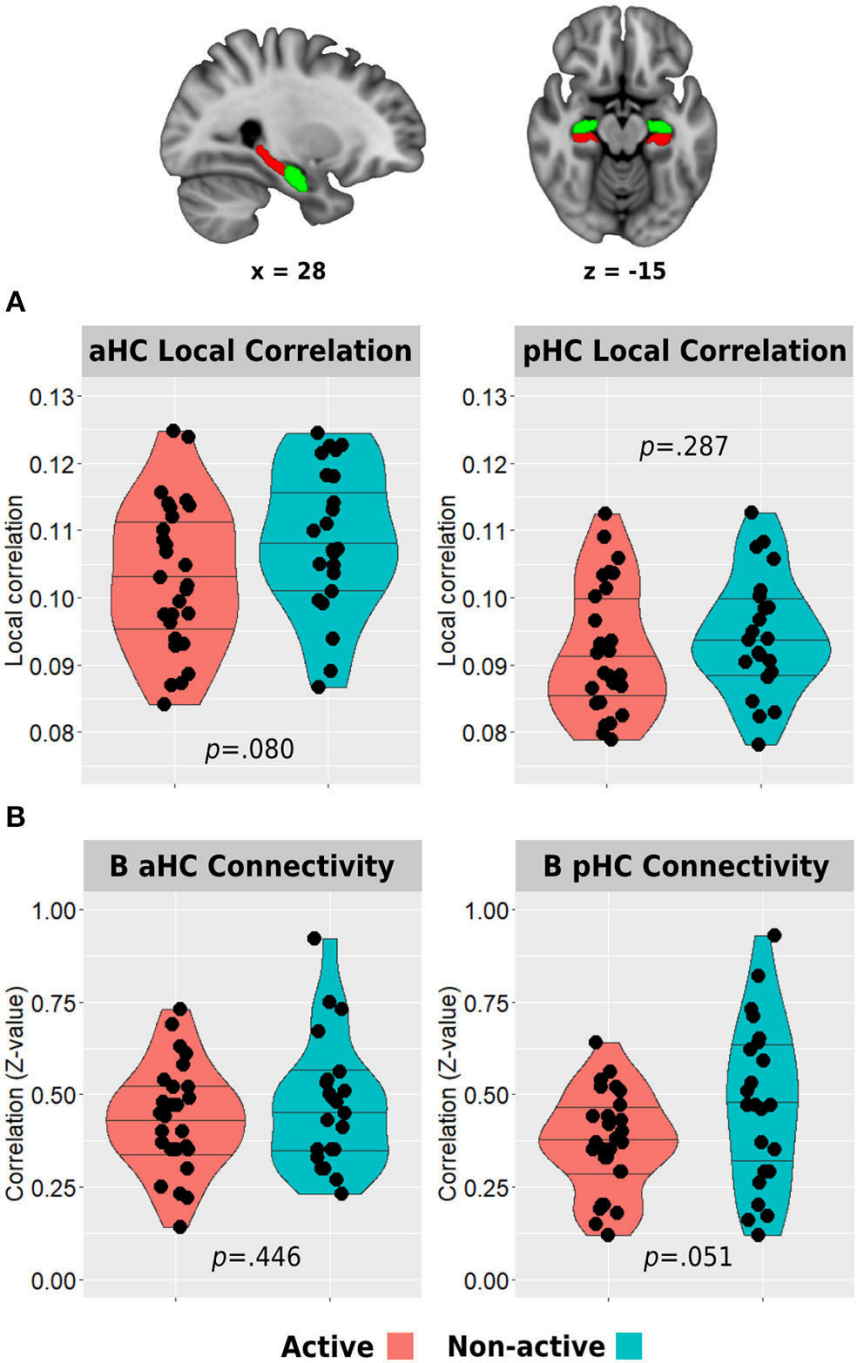
Between-group differences in hippocampal resting-state local correlation **(A)** and between-hemisphere anterior and posterior hippocampal resting-state functional connectivity **(B)**. The top row demonstrates the anterior (green) and posterior (red) hippocampal masks used in the analyses. Horizontal lines in between-group plots represent 25, 50, and 75% quantiles. B, bilateral; aHC, anterior hippocampus; pHC, posterior hippocampus.

Resting-state functional connectivity was carried out using the functional connectivity toolbox *CONN* v.19c (nitrc.org/projects/conn). Preprocessing included discarding the first five TRs to allow steady-state magnetization. Functional images were slice-time corrected, realigned to the middle volume, motion-corrected, and normalized to the standard MNI152 space. Spatial smoothing was performed using a 5-mm FWHM Gaussian kernel. To reduce noise, functional volumes were band-pass filtered at 0.008–0.15, and the component-based method (CompCor) was used to extract noise signals (e.g., white matter, cerebrospinal fluid (CSF), and movement artifact) that were used as nuisance regressors to denoise the data. In addition, images that were regarded as movement outliers were regressed out. Movement outlier volumes were detected using the ART toolbox (nitrc.org/projects/artifact_detect/) and defined as volumes with a movement >0.9 mm or signal intensity changes >5 SD. These volumes were also used as nuisance regressors at the denoising step. No participant demonstrated more than 30% of removed volumes. One participant from the active group did not go through the resting-state run. Remote functional connectivity of the anterior and posterior hippocampi with the main hubs of the DMN was examined by creating specific masks of these regions of interest. Anterior and posterior hippocampal masks were again created from https://neurovault.org/collections/3731/ (Figure 3A). To create the DMN masks, we used a deactivation contrast in the memory encoding task to elicit areas demonstrating decreased activity during the task (Supplementary Table 1). The two main and larger clusters revealed from this analysis were anatomically corresponding to the posteromedial and medial prefrontal cortices and were used to create the masks for the hippocampal-DMN analyses. The mean *z*-transformed correlation value between each pair of these eight areas was then used to examine differences in hippocampal-DMN functional connectivity between the groups. Intra-hippocampal resting-state connectivity was examined in two ways. First, we computed the *z*-transformed correlation between the time series of bilateral anterior hippocampi and bilateral posterior hippocampi. Second, we calculated the local correlation within each hemispheric anterior or posterior region using the local correlation implemented in *CONN*. This index represents a measure of local coherence at each voxel, characterized by the strength and sign of connectivity between a given voxel and the neighboring regions in the brain (Deshpande et al., 2009). We used a 6-mm kernel for the Gaussian weighting function characterizing the size of the local neighborhoods.

### General Cognitive Evaluation

All participants were evaluated for general cognitive functioning and were screened for objective general cognitive decline using the Montreal Cognitive Assessment (MoCA) (Oren et al., 2015).

### Statistical Analysis

Statistical analyses and visualizations were performed and constructed using IBM SPSS Statistics Version 24.0 (Armonk, NY: IBM Corp.) and R Version 4.0.3 (R Core Team, Vienna, Austria; https://www.R-project.org/). Between-group differences were tested using non-parametric permutation testing. These procedures were conducted with 10,000 iterations, permuting each hippocampal or memory measure, and preserving the original group sizes. Then, the observed between-group mean difference in each measure was compared with all between-group differences obtained from the permuted null distribution. The *p*-values were determined as the probability of getting equal or greater between-group differences based on the null distribution. Correlations between Vo_2_peak and hippocampal and memory measures were evaluated using non-parametric partial Spearman's rank correlations controlling for age, sex, and years of education. Since the implemented recognition task has no well-established norms, a linear regression was used to create standardized residuals of the memory scores controlled for age, sex, and education, which was used as the memory performance measure for further analyses. In addition, since the local correlation of bilateral anterior or posterior hippocampi was highly correlated between the homologous regions, we used the average local correlation value of the bilateral anterior/posterior hippocampal regions for further analyses. Between-group differences in continuous background characteristics (i.e., age, education, and MoCA) were evaluated using the Wilcoxon–Mann–Whitney *U*-test. Between-group differences in the proportions between males and females, marital status, smoking status, and hypertension diagnosis were assessed using the chi-squared test. As only two participants reported being currently smoking, we characterized the participants as “*current or past smokers*” and “*non-smokers*.” *p*-values of hippocampal-DMN connectivity between-group tests and correlations (8 tests each) were corrected for multiple comparisons using the false discovery rate (FDR) method (Benjamini and Hochberg, 1995).

## Results

### Study Participants

The active and non-active groups were not statistically different in any background characteristics, including years of education, general cognitive functioning, smoking status, and hypertension diagnosis (*p* > 0.05; Table 1).

**Table 1:** General socio-demographic and cognitive characteristics of participants (means ± SD).

**Variable**	**Whole sample**	**Aerobically active**	**Non-Active**	**Between-Group**
	**(*n* = 52)**	**(*n* = 29)**	**(*n* = 23)**	***p*-value**
Age (years)	70.83 ± 3.9	70.34 ± 4.0	71.43 ± 3.7	0.247
Sex (female/male)	22/30	11/18	11/1	0.473
Education (years)	15.84 ± 3.3	16.17 ± 3.7	15.41 ± 2.8	0.644
Marital status (married/unmarried)	45/7	26/3	19/4	0.460
Smoking (current or past smokers/non-smokers)	25/27	14/15	11/12	0.974
Hypertension (diagnosed/undiagnosed)	9/43	3/26	6/17	0.136
MoCA (raw score)	24.81 ± 2.5	25.13 ± 2.2	24.39 ± 2.8	0.349

*MoCA, Montreal Cognitive Assessment; SD, standard deviation*.

### Aerobic Exercise Habits and Vo_2_Peak Characteristics

The report of study participants of weekly aerobic activity ranged from being completely sedentary (not engaging in exercise-oriented activities, *n* = 18) to 6 days per week (*n* = 3). Seventeen participants reported exercising 3 times per week. Exercising on a single day (*n* = 5), twice a week (*n* = 3), 4 times per week (*n* = 4), and 5 times (*n* = 2) were also reported. The active group demonstrated statistically significant higher weekly frequency of exercise sessions and Vo_2_peak values (Table 2).

**Table 2:** Aerobic activity and fitness characteristics of participants (means ± SD).

**Variable**	**Whole sample**	**Aerobically active**	**Non-Active**	**Between-Group**
	**(*n* = 52)**	**(*n* = 29)**	**(*n* = 23)**	***p*-value**
Aerobic exercise (d/week)	2.04 ± 1.8	3.48 ± 1.1	0.22 ±.4	<0.001
Vo_2_peak (ml/kg/min)	25.25 ± 8.3	30.58 ± 6.2	18.86 ± 3.8	<0.001

*d/week, days per week; kg, kilogram; min, minute; ml, milliliter; SD, standard deviation; Vo_2_peak, peak oxygen consumption*.

### Hippocampal Activation

#### Between-Group Differences

Whole-brain analysis of all participants revealed bilateral anterior hippocampal activation during the associative memory encoding task (Figure 2A). Both groups demonstrated a positive mean activation level in both right and left anterior hippocampi during the task. However, the active group activation levels were significantly lower compared with the non-active group (right anterior hippocampus: 0.09 ± 0.31 vs. 0.38 ± 0.31, *p* = 0.002; left anterior hippocampus: 0.13 ± 0.31 vs. 0.33 ± 0.32, *p* = 0.027; Figure 2B).

#### Association With Vo_2_Peak

**Figure 4:**
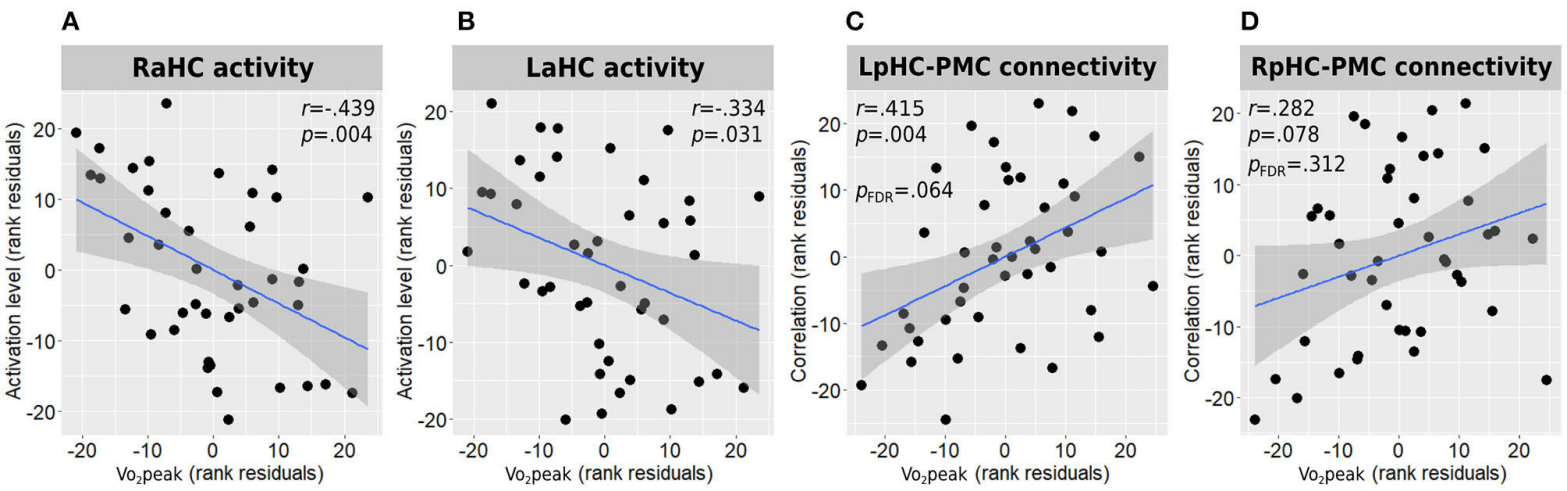
Associations between Vo_2_peak and hippocampal function. Correlations between Vo_2_peak and right **(A)** and left **(B)** anterior hippocampal activity during memory encoding and with the connectivity strength of the left **(C)** and right **(D)** posterior hippocampi connectivity with the posteromedial cortex are presented. All correlations are adjusted for age, sex, and education. L/RaHC, left/right anterior hippocampus; L/RpHC, left/right posterior hippocampus; PMC, posteromedial cortex; Vo_2_peak, peak oxygen consumption.

In accordance with the between-group results, higher Vo_2_peak was negatively correlated with both right and left anterior hippocampal activation levels during the memory encoding task [*r*(38) = −0.439, *p* = 0.004 and *r*(38) = −0.334, *p* = 0.031, respectively] (Figures 4A,B).

### Distant Hippocampal Resting-State Functional Connectivity

#### Between-Group Differences

**Figure 5:**
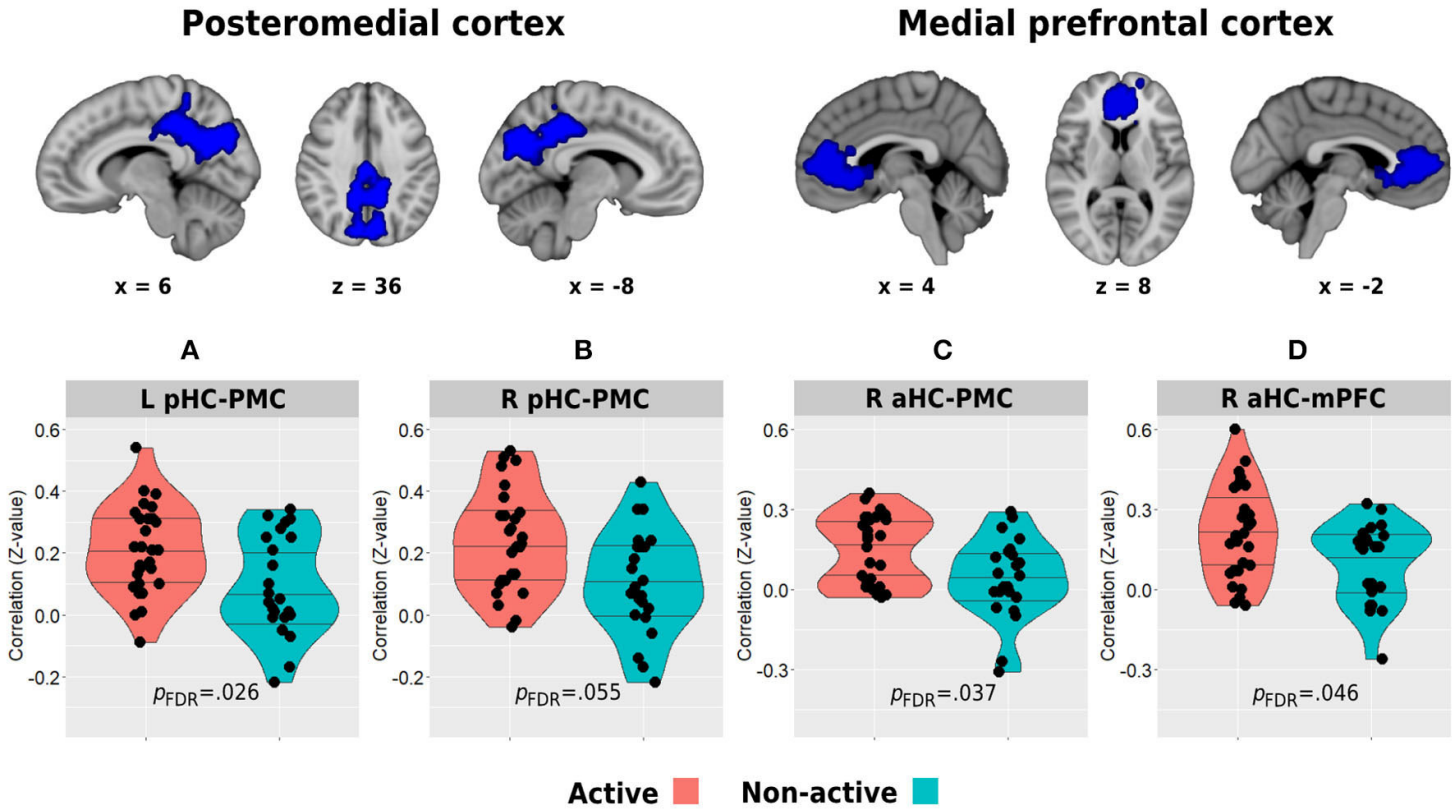
Between-group differences in resting-state functional connectivity of the posterior **(A,B)** and anterior **(C,D)** hippocampi with the posteromedial and medial prefrontal cortical clusters which demonstrated decreased activity during the memory encoding paradigm. The top row demonstrates the DMN clusters of interest. Horizontal lines in between-group plots represent 25, 50, and 75% quantiles. aHC, anterior hippocampus; pHC, posterior hippocampus; mPFC, medial prefrontal cortex; PMC, posteromedial cortex; L, left; R, right.

Whole-brain analysis of deactivation contrast during memory encoding revealed several clusters of areas demonstrating decreased activity which are usually attributed to the DMN (Supplementary Table 1). The two main and larger clusters observed corresponded to the posteromedial and medial prefrontal cortices, which were used as the regions of interest for the hippocampal-DMN resting-state functional connectivity analysis (Figure 5). In turn, between-group analysis revealed both the left and right posterior hippocampi to demonstrate higher functional connectivity with the posteromedial cortex in the physically active group compared with the non-active group (left posterior hippocampus: 21 ± 0.14 vs. 0.09 ± 0.16, *p* = 0.009, *p*FDR = 0.026; right posterior hippocampus: 23 ± 0.16 vs. 0.11 ± 0.17, *p* = 0.007, *p*FDR = 0.055; Figures 5A,B). In addition, the aerobically active group demonstrated higher functional connectivity between the right anterior hippocampus and both DMN hubs (posteromedial: 0.15 ± 0.13 vs. 0.04 ± 0.15, *p* = 0.009, *p*FDR = 0.037; medial prefrontal: 21 ± 0.17 vs. 0.10 ± 0.15, *p* = 0.023, *p*FDR = 0.046; Figures 5C,D). All other hippocampal-DMN regional connectivity values were not different between the groups (*p* > 0.40).

#### Association With Vo_2_Peak

Higher Vo_2_peak was found to demonstrate a moderate positive correlation with the connectivity strength of the left posterior hippocampus and the posteromedial cortex [*r*(38) = 0.415, *p* = 0.004, *p*FDR = 0.064; Figure 4C] and a weak-to-moderate positive correlation with the connectivity strength of the right posterior hippocampus and the posteromedial cortex which demonstrated a trend toward statistical significance before multiple testing correction [*r*(38) = 0.282, *p* = 0.078, *p*FDR = 0.312; Figure 4D]. All other hippocampal-DMN connectivity values demonstrated weak to negligible correlations.

### Local Hippocampal Resting-State Functional Connectivity

#### Between-Group Differences

The active group demonstrated lower resting-state local correlation in both anterior and posterior hippocampi, but only the anterior hippocampus reached a trend for statistical significance (anterior: 0.103 ± 0.011 vs. 0.108 ± 0.011, *p* = 0.080; posterior: 0.092 ± 0.010 vs. 0.094 ± 0.009, *p* = 0.287; Figure 3A). In addition, the resting-state connectivity of the bilateral posterior hippocampus was lower in the active group compared with the non-active group (0.37 ± 0.14 vs. 0.47 ± 0.22, *p* = 0.051). No difference was found between the bilateral anterior hippocampus connectivity across the groups (0.43 ± 0.14 vs. 0.47 ± 0.17, *p* = 0.446; Figure 3B).

#### Association With Vo_2_Peak

Peak oxygen consumption (Vo_2_peak) demonstrated weak correlations with anterior and posterior intra-hippocampal local correlations [*r*(38) = −0.232, *p* = 0.150 for both regions] and with inter-hemispheric hippocampal connectivity [anterior: *r*(38) = 0.089, *p* = 0.585; posterior: *r*(38) = −0.166, *p* = 0.305].

### Memory Performance and Relationship With Hippocampal Activation During the Task

**Figure 6:**
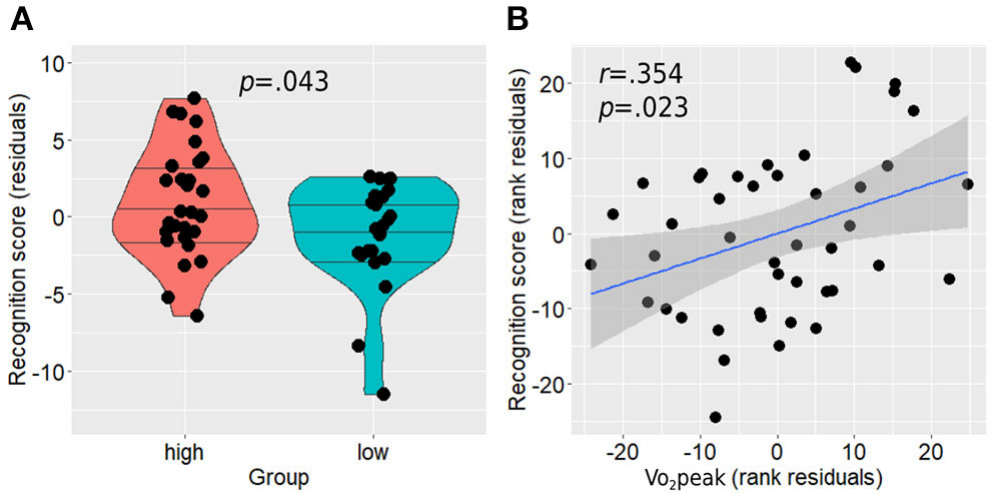
Memory recognition performance differences between the groups **(A)** and its association with Vo_2_peak while controlling for age, sex, and education **(B)**. Horizontal lines in between-group plots represent 25, 50, and 75% quantiles. Vo_2_peak, peak oxygen consumption.

Between-group analysis revealed a statistically significant difference between the groups with the active group demonstrating higher performance on the memory recognition task compared with the non-active group (residual score 0.24 ± 0.95 vs. −0.30 ± 0.93, *p* = 0.043; Figure 6A). In addition, higher Vo_2_peak was positively associated with higher performance in the recognition task [*r*(39) = 0.354, *p* = 0.023; Figure 6B]. No correlation was found between anterior hippocampal activity level and memory performance on the whole sample level in both right [Spearman rho(45) = −0.139, *p* = 0.350] and left [Spearman rho(45) = −0.199, *p* = 0.180] hippocampi while controlling for age, sex, and education, or when examined separately within each group for the active [right: Spearman rho(24) = 0.009, *p* = 0.967, left: Spearman rho(24) = 0.009, *p* = 0.964] and non-active [right: Spearman rho(16) = 0.156, *p* = 0.536, left: Spearman rho(16) = −0.261, *p* = 0.296] groups.

## Discussion

As aging has been demonstrated to be associated with compromised brain structure and function (Raz et al., 2005; Mormino et al., 2012; Nobis et al., 2019; Nyberg et al., 2019), a study has been conducted to investigate lifestyle factors that may attenuate this deterioration with advanced age (Akbaraly et al., 2009; Foubert-Samier et al., 2012). Physical exercise, aerobic in particular, has been associated with a reduced risk of cognitive decline and dementia (Rovio et al., 2005) and hippocampal resilience in older adults (Erickson et al., 2011; Maass et al., 2015; Sexton et al., 2016). However, studies investigating the neuroprotective relationship between physical activity and hippocampus have been mainly focusing on hippocampal structure (Sexton et al., 2016) and to a lesser extent on hippocampal cerebrovascular properties (Zimmerman et al., 2014; Maass et al., 2015). This study aimed to broaden this line of research by investigating the relationship between aerobically active lifestyle and hippocampal function in cognitively intact older adults using both resting-state and task-based fMRI experiments. In addition, we aimed to examine whether Vo_2_peak, a potential mediator of aerobic exercise effects, may be associated with hippocampal functional characteristics in a dose-response manner.

While anterior hippocampal activity patterns during memory encoding have been generally demonstrated to exhibit age-related decline, some older individuals have been shown to exhibit increased hippocampal activity, or hyperactivity, exceeding values demonstrated in even younger counterparts. In this study, both active and non-active groups demonstrated increased bilateral anterior hippocampal activation during the associative memory task. However, the non-active group demonstrated a significantly higher activity level compared with the active group, with this difference being more pronounced in the right anterior hippocampus. In accordance with this trend, Vo_2_peak was also negatively correlated with bilateral anterior hippocampus activation level. Mormino et al. (2012) found successful episodic memory encoding to be associated with right hippocampal hyperactivity in cognitively normal older adults with high Aβ burden compared with young adults and older adults without significant AD pathology. While not reporting on the specific relationship between the hippocampal hyperactivity and post-task memory performance, they demonstrated that the general increase in brain activity observed in the high-risk participants was associated with favorable memory performance, suggesting for a compensatory mechanism as being previously shown in patients with amnestic MCI (Dickerson et al., 2005; Eisenstein et al., 2020). However, this explanation was ruled out in the current cognitively intact sample, since the increased hippocampal activity was not associated with better memory performance in each group separately or at the whole sample level. In fact, previous studies supported the possibility that increased hippocampal activity may reflect an aberrant dysfunctional activity pattern. Nyberg et al. (2019) found older adults exhibiting right anterior hippocampal hyperactivity to demonstrate lower memory performance, higher genetic risk of AD, and higher incidence of deteriorating to dementia at follow-up. Leal et al. (2017) found increased right hippocampal activity during memory encoding to be associated with longitudinal accumulation of Aβ several years later, which in turn was associated with steeper memory decline. Importantly, this association was not demonstrated for other cortical areas demonstrating increased activity prior to follow-up. Hippocampal hyperactivity during different types of memory encoding tasks was also shown to be associated with increased tau pathology and lower memory performance in cognitively normal older adults (Berron et al., 2019; Huijbers et al., 2019). Further, the study supporting the potential link between AD pathology and hippocampal hyperactivity comes from the findings in genetic models of the disease. Aberrant hyper-excitatory activity in the hippocampus is a consistent finding in animal models of AD (Palop et al., 2007; Haberman et al., 2017), whereas young adults carrying the mutation for the familial subtype of AD have been repeatedly demonstrating right anterior hippocampal hyperactivity during memory encoding compared with non-carrier controls (Quiroz et al., 2010; Reiman et al., 2012). Furthermore, increased hippocampal activity during memory encoding has also been shown in young individuals at risk for late-onset sporadic AD, i.e., APOE ε4 carriers (Filippini et al., 2009; Dennis et al., 2010). The observed relationship between hippocampal hyperactivity and Aβ accumulation may be a reflection of a vicious neuropathological cycle in which over-excitatory neurons drive local Aβ aggregation, which, in turn, further increases neuronal excitability levels (Palop et al., 2007; Bero et al., 2011). While aerobic exercise has been repeatedly demonstrated to be associated with a lower risk of AD and dementia (Rovio et al., 2005; Larson et al., 2006; Andel et al., 2008), a mechanism that may link aerobic exercise with hippocampal hyper-excitability and dysfunction attenuation in aging may lie in Aβ metabolism. AD model mice demonstrated lower hippocampal amyloid plaque load following exercise intervention, with greater training intensity resulting in lower Aβ burden (Thomas et al., 2020). Furthermore, evidence for increased glymphatic clearance of hippocampal amyloid plaques in aged mice was also demonstrated following a single session of aerobic exercise (He et al., 2017). A study in already diagnosed human patients with AD did not find the aerobic intervention to affect Aβ levels; however, the late clinical stage of the participants on the AD continuum, the relatively short duration of the intervention, and significantly higher Aβ levels in the exercise group at baseline may account for the lack of interventional efficacy observed in this study (Frederiksen et al., 2019). In addition, while normal hippocampal function requires a balance between excitation and inhibition, abnormal hippocampal excitation in pathological aging has also been linked to GABAergic inhibitory dysfunction (Hazra et al., 2013; Tong et al., 2014). Aerobic exercise, in turn, has been demonstrated to induce neuroprotective inhibitory modulation in the hippocampus of hyper-excitatory epileptic rats (Lim et al., 2015; Barzroodi Pour et al., 2019). This, in turn, suggests another potential neurobiological mechanism that may underlie the relationship between aerobic exercise and attenuated hippocampal hyperactivity observed in this study.

The second primary finding in this study is the differences observed in hippocampal resting-state connectivity between the groups. Namely, aerobically active lifestyle (and to a lesser extent Vo_2_peak) was found to be associated with higher distant hippocampal functional connectivity with core hubs of the DMN, i.e., the posteromedial and medial prefrontal cortices and with lower local intra-hippocampal connectivity. The hippocampus is a part of the medial temporal subsystem of the DMN (Andrews-Hanna et al., 2010), and these regions have been shown to functionally cooperate in cortico-hippocampal memory networks (Ranganath and Ritchey, 2012; Cooper and Ritchey, 2019). Previous studies demonstrated decreased cortico-hippocampal connectivity in healthy older adults. While Damoiseaux et al. (2016) found only the posterior hippocampus to demonstrate an age-related decline in functional connectivity with the DMN, Salami et al. (2014) demonstrated this age-related effect to occur in both anterior and posterior hippocampal subparts, and that higher connectivity values were positively correlated with episodic memory performance. We found a link between the physically active lifestyle and higher connectivity of the bilateral posterior hippocampus with the posteromedial cortex while also higher connectivity of the right anterior hippocampus with both posteromedial and medial prefrontal cortices. In addition to higher distant connectivity, the active group in our study also demonstrated a trend toward lower intra-hippocampal connectivity expressed as lower local correlations within the anterior hippocampus and between the posterior hippocampi across the two hemispheres. Higher within- and between-hippocampus correlations have been previously demonstrated with increasing age, negatively correlating with memory performance, and load of AD pathology (Salami et al., 2014; Harrison et al., 2019). Harrison et al. (2019) further demonstrated that increased local within hippocampal correlation, represented by higher regional homogeneity, was associated with decreased cortico-hippocampal connectivity, suggesting that both age and AD pathology may be associated with increasing hippocampal functional disconnection and isolation. In contrast, our findings provide evidence that aerobic activity and physically active lifestyle may constitute a neuroprotective factor in face of this aspect of hippocampal dysfunction. In turn, these results extend previous studies which demonstrated a relationship between physical activity and increased DMN connectivity (Voss et al., 2010; Boraxbekk et al., 2016). Reduced functional connectivity of the DMN is associated not only with aging (Salami et al., 2014) but also with early biomarkers of AD pathology, as being highly sensitive regions to early Aβ accumulation (Palmqvist et al., 2017). Given the evidence pointing at the potential role that aerobic activity may have in increasing the glymphatic removal of Aβ deposition from the interstitial space, it may underlie, at least partially, the observed relationship with higher functional connectivity of the two core hubs of DMN with the hippocampus. In addition, one of the hallmarks of exercise-induced mechanisms observed in animal models is the upregulation of brain and hippocampal brain-derived neurotrophic factor (BDNF) (Oliff et al., 1998; Vaynman et al., 2004; Berchtold et al., 2005). Among its versatile functions, BDNF promotes neurite outgrowth and synaptogenesis, plays a significant role in synaptic plasticity, and is involved in learning and memory processes (Binder and Scharfman, 2004; Leal et al., 2015). In human older adults, increased serum levels of BDNF were associated with increased parahippocampal functional connectivity following 1 year of aerobic intervention, supporting the potential role of BDNF as a neurobiological mediator of exercise-induced changes in brain function (Voss et al., 2013). It is important to note that although Vo_2_peak was associated with some of the hippocampal measures we examined in this study, it did not explain all the between-group differences observed, especially in terms of distant and local hippocampal connectivity. Although it may support a potential mediating role of Vo_2_peak in some of the exercise-related hippocampal function effects, it also suggests that other potential mechanisms play a role in mediating this relationship.

This study adds evidence regarding the functional neuroprotective potential of aerobic exercise which is in line with previous models that aim to explain neurocognitive differences in older adults. The ability to successfully perform a cognitive task while recruiting less neural resources, or increased neural efficiency, has been suggested to constitute a mechanism, which may underlie the superior cognitive performance observed in some older individuals compared with others (Barulli and Stern, 2013). Increased neural efficiency has been previously shown to be associated with favorable cognitive performance in aging (Steffener et al., 2011; Hakun et al., 2015) and was also demonstrated to be expressed in healthy older adults compared with neurodegenerative patients (Sole-Padulles et al., 2009). In this study, since the physically active group also demonstrated higher memory performance, in addition to the observed lower hippocampal activation, these findings may suggest that these individuals may be able to use more efficiently hippocampal resources in order to cope with memory demands.

### Study Limitations

The main limitation of this study lies in its cross-sectional nature. Although previous evidence from animal models and human participants support the observed relationship between aerobic exercise and hippocampal function from a neurobiological mechanistic point of view, we cannot state that the physically active lifestyle is, in fact, the causal mediator of the attenuation in hippocampal dysfunction observed in the current sample of cognitively intact older adults. Instead, these results should serve as a starting point for future longitudinal and interventional studies that are needed to explore the time-dependency between aerobic exercise and functional adaptations of the hippocampus in human aging and to establish a causal effect. Another issue that should be kept in mind is that other factors that have not been evaluated in this study could potentially contribute to the explanation of the relationship observed. These include, but are not limited to, differences in genetic background and latent pathological processes. Although we excluded participants with unbalanced medical conditions, this population is prone to age-related pathologies, and it is possible that at least some of the included participants had ongoing pathological processes that are still not symptomatically expressed and therefore they were not aware of.

## Conclusion

While the relationship between physical activity and hippocampal function in human aging has been sparsely investigated, this study provides evidence for a potential relationship between lifestyle habits of aerobic exercise and attenuation of different aspects of hippocampal dysfunction, normally observed in older individuals, namely alteration in patterns of hippocampal activity and connectivity. By that, the current research extends previous studies demonstrating the relationship between physical activity and aging brain in general and specifically the neuroprotective effect of aerobic exercise on the aged hippocampus. In addition, our results suggest that although Vo_2_peak may be associated with some aspects of hippocampal function in older adults, other factors may also contribute to the relationship between aerobic exercise and distinct hippocampal functions.

## Data Availability Statement

Due to medical confidentiality and since participants did not consent to having their data publicly published, the unidentified data (e.g., data spreadsheet) and code that support the findings of this study are available from the corresponding author without undue reservation.

## Ethics Statement

The studies involving human participants were reviewed and approved by Human Studies Committee of Tel Aviv Sourasky Medical Center. The patients/participants provided their written informed consent to participate in this study.

## Author Contributions

TE: conceptualization, investigation, methodology, software, formal analysis, writing—original draft, writing—review & editing, project administration, and visualization. NG: supervision and conceptualization. TH: resources and funding acquisition. OH: project administration. YL: conceptualization, methodology, supervision, writing—review & editing, project administration, resources, and funding acqusition. All authors contributed to the article and approved the submitted version.

## Funding

This study has been funded from the grant of the Israel Science Foundation (ISF) provided to YL (Grant No. 1573/18) and the financial support of the Sagol Family Foundation for Brain Research provided to Sagol Brain Institute. The funding sources were not involved in the conduction of the research.

## Conflict of Interest

The authors declare that the research was conducted in the absence of any commercial or financial relationships that could be construed as a potential conflict of interest.

## Publisher's Note

All claims expressed in this article are solely those of the authors and do not necessarily represent those of their affiliated organizations, or those of the publisher, the editors and the reviewers. Any product that may be evaluated in this article, or claim that may be made by its manufacturer, is not guaranteed or endorsed by the publisher.
