# Supplementary material for: Physically Active Lifestyle Is Associated With Attenuation of Hippocampal Dysfunction in Cognitively Intact Older Adults
Source: Front Aging Neurosci. 2021 Oct 6;13:720990. doi: 10.3389/fnagi.2021.720990 (PMC8527880; doi:10.3389/fnagi.2021.720990)
Supplement: Supplementary file 1 [file Table_1.DOCX]

Table S1. Significant clusters demonstrating deactivation during memory encoding (cluster-corrected).

| Cluster | *x* | *y* | *z* | Cluster  Size | z-value  max |
| --- | --- | --- | --- | --- | --- |
| 1 | 4 | -30 | 44 | 4308 | 5.26 |
| 2 | 0 | 42 | -4 | 2701 | 4.77 |
| 3 | -40 | -74 | 40 | 1523 | 4.86 |
| 4 | 66 | -20 | -4 | 1276 | 4.91 |
| 5 | 46 | -74 | 38 | 960 | 4.73 |
| 6 | 34 | -52 | 4 | 682 | 5.06 |
| 7 | -30 | -58 | 4 | 568 | 4.73 |

Coordinates are in MNI space
